# Left Atrial Appendage Thrombosis and Persistent Atrial Fibrillation: combined Treatment with a Totally Thoracoscopic Approach

**DOI:** 10.21470/1678-9741-2020-0028

**Published:** 2020

**Authors:** Igor Vendramin, Andrea Lechiancole, Luca Rebellato, Ermanno Dametto, Uberto Bortolotti, Ugolino Livi

**Affiliations:** 1 Cardiothoracic Department, University Hospital of Udine, Udine, Italy.; 2 Division of Cardiology, Civic Hospital, Pordenone, Italy.

**Keywords:** Pulmonary Veins, Atrial Fibrilation, Atrial Appendage, Follow-up Studies, Catheter Ablation, Thromboembolism, Thrombosis, Longitudinal Studies

## Abstract

Minimally invasive surgical ablation is generally contraindicated in patients with atrial fibrillation and thrombosis of the left atrial appendage.

We have treated three of these patients using an innovative technique based on a bilateral video-thoracoscopic approach, performing a continuous encircling lesion at the pulmonary veins outflow with radio-frequency ablation, simultaneously excluding the left atrial appendage. The postoperative course was uneventful, without neurologic events and all patients maintained a stable sinus rhythm at 1-year follow-up.

This procedure represents a new mini-invasive method to treat persistent atrial fibrillation when partial thrombosis of the left atrial appendage contraindicates other ablation techniques.

**Table:** 

Abbreviations, acronyms & symbols	
AF	= Atrial fibrillation
LAA	= Left atrial appendage
TEE	= Transesophageal echocardiography

## INTRODUCTION

In patients with persistent atrial fibrillation (AF) and left atrial appendage (LAA) thrombosis, current ablation techniques are contraindicated^[1]^. We report a new mini-invasive surgical approach that has been shown to be effective in the treatment of both AF and LAA thrombosis by restoring sinus rhythm and avoiding potentially severe neurological complications.

## TECHNIQUE

**Fig. 1:**
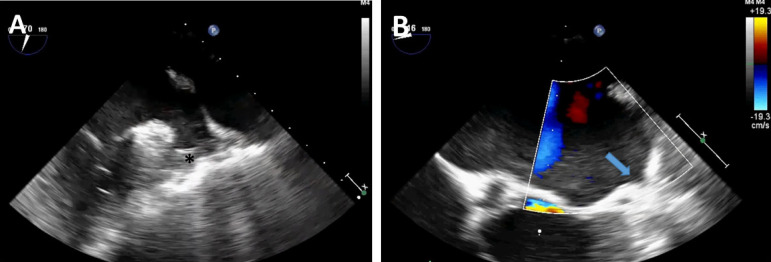
A) Intraoperative transesophageal echocardiography showing left atrial thrombus (asterisk); B) Postoperative result with left atrial appendage exclusion (arrow).

Under general anaesthesia, tracheal intubation is performed to allow selective lung ventilation. Transoesophageal echocardiography (TEE) monitoring is of paramount importance to confirm or detect any LAA thrombus (Figure 1) and is maintained throughout the procedure. Then, three port-access sites on the right chest are identified: in the 3^rd^ intercostal space (anterior axillary line) for the camera and CO_2_ insufflation and in the 2^nd^ and 5^th^ intercostal spaces for instrumentation. In the left hemithorax (mid-axillary line), three similar ports are marked.

**Fig. 2:**
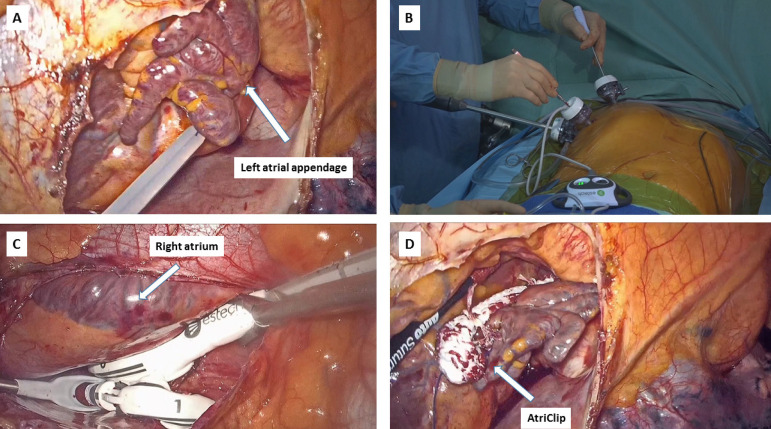
A) Base of the left atrial appendage encircled and snared with 3/0 Prolene Ethibinder; B) Operative instrumentation and surgical approach; C) Creation of the box lesion around the pulmonary veins; D) Final result after AtriClip implantation at the base of the left atrial appendage.

The left hemithorax is approached through 3 ports using 12-mm skin incisions. The pericardium is opened and exposed with sutures. The base of the LAA is encircled and snared with 3/0 Prolene Ethibinder (Johnson & Johnson^®^, New Jersey, USA) (Figure 2A), which is an endo-suture system with a pre-assembled loop used to stabilize the thrombus. Subsequently, during single left lung ventilation, the right hemithorax is approached (Figure 2B), the pericardium opened and exposed. Blunt dissection with Endopeanuts (US Surgical, Norwalk, CT) is performed between the superior vena cava and the right superior pulmonary vein, reaching the transverse sinus. The oblique sinus is exposed by dissecting the inferior vena cava from the right inferior pulmonary vein. Two flexible components of the introducer (Atricure Inc., Mason, OH, USA) are passed through the oblique and transverse sinuses around the pulmonary veins and pulled until their magnetic tips meet. Then the Atricure Cobra Fusion^TM^ ablation probe is connected to the introducer and advanced inside the chest to create the “box lesion” (Figure 2C); the ablation catheter is stabilized by suction, ensuring a temperature-controlled use of monopolar and bipolar energy sources. Multiple lesions are performed (60 seconds at 70°C each) moving the catheter circumferentially to treat all underlying structures. At this stage, external electrical cardioversion is performed whenever the sinus rhythm is not regained; any exit block at this stage is assessed by pacing the right pulmonary veins. Finally, through the left hemithorax, the base of LAA is measured with the dedicated sizer and an Atricure AtriClip Pro II is introduced through the most caudal port. Under TEE and visual control, the clip is implanted at the LAA base (Figure 2D), while exposure is facilitated by gentle traction on Ethibinder. The accesses are closed, after partial pericardium suturing and chest tubes placement.

## DISCUSSION

Patients with persistent AF are currently treated by various ablation techniques with rewarding results^[2-4]^. However, the presence of LAA thrombosis still represents a major issue due to the potential risks of systemic embolization once sinus rhythm is restored. In this setting, we have adopted a new totally thoracoscopic approach that combines initial snaring of the LAA base through the left hemithorax, followed by radiofrequency ablation after entering the right chest. Three patients with persistent AF and LAA thrombosis were treated with this approach. Two of them were male and one female, 60, 67 and 70 years old. The postoperative course was uneventful, without neurologic events; all were discharged within one week and maintained a stable sinus rhythm at 1-year follow-up.

Although our experience is limited, we believe that ideal candidates should be patients with persistent AF and partial LAA thrombosis, which represents a contraindication to both endovascular and surgical ablation. The most favourable anatomy of LAA for adequate exclusion with an AtriClip is most likely represented, in our opinion, by the chicken wing and windsock morphological patterns, but we believe that, with increasing experience, almost all types of LAA might be successfully treated. Furthermore, an important prerequisite is also represented by the intra-atrial extension of the thrombus, which should not involve the LAA base, preventing the safe application of the snare.

The presence of thrombus in a fragile structure such as LAA requires special care in management and manipulation. Stabilization of the thrombus with a temporary closure of the LAA base with a 3/0 Prolene Ethibinder loop allowed to achieve a box lesion ablation and subsequently allowed to complete its exclusion with an AtriClip without removing the loop. The efficacy of LAA exclusion by AtriClip implantation has been recently validated by angiographic studies^[5,6]^. Finally, we believe that another important issue is represented by the anticoagulant regime that should be adopted before the procedure. All of our patients where kept on oral anticoagulation with warfarin for at least 6 months prior to ablation; we believe that a more prolonged treatment (i.e., for more than 12 months) is not advisable since an excessive delay in surgical timing could minimize the benefits of the procedure leading to persistent AF. Furthermore, we can exclude that in the meantime the thrombus has dissolved with anticoagulation, which would allow to switch to a transcatheter procedure and, after 6 months, we can be reasonably sure that the thrombus is more stable within the LAA, reducing the risk of fragmentation during the procedure.

## CONCLUSION

The procedure herein described represents a new innovative and feasible single-stage mini-invasive method which allows to successfully treat persistent AF and simultaneously exclude a thrombosed LAA with AtriClip, eliminating the potential risks of thrombus fragmentation and migration with possible harmful systemic embolization.

**Table:** 

**Authors' roles & responsibilities**	
IV	Substantial contributions to the conception of the work; or the acquisition, analysis, or interpretation of data for the work
AL	Substantial contributions to the conception or design of the work; or the acquisition, analysis, or interpretation of data for the work
LR	Substantial contributions to the conception or design of the work; or the acquisition, analysis, or interpretation of data for the work
ED	Substantial contributions to the conception or design of the work; or the acquisition, analysis, or interpretation of data for the work
UB	Substantial contributions to the conception or design of the work; or the acquisition, analysis, or interpretation of data for the work
UL	Substantial contributions to the conception or design of the work; or the acquisition, analysis, or interpretation of data for the work; final approval of the version to be published
